# Superdominant Right Coronary Artery with Absence of Left Circumflex and Anomalous Origin of the Left Anterior Descending Coronary from the Right Sinus: An Unheard Coronary Anomaly Circulation

**DOI:** 10.1155/2015/721536

**Published:** 2015-07-09

**Authors:** Marcos Danillo Peixoto Oliveira, Fernando Roberto de Fazzio, José Mariani Junior, Carlos M. Campos, Luiz Junya Kajita, Expedito E. Ribeiro, Pedro Alves Lemos

**Affiliations:** Department of Interventional Cardiology, Heart Institute(InCor) of the University of São Paulo, Avenida Dr. Enéas de Carvalho Aguiar 44, 05403-900 São Paulo, SP, Brazil

## Abstract

Coronary artery anomalies are congenital changes in their origin, course, and/or structure. Most of them are discovered as incidental findings during coronary angiographic studies or at autopsies. We present herein the case of a 70-year-old man with symptomatic severe aortic valvar stenosis whose preoperative coronary angiogram revealed a so far unreported coronary anomaly circulation pattern.

## 1. Introduction

Coronary artery anomalies (CAA) are a diverse group of congenital disorders, and the pathophysiological mechanisms and manifestations are highly variable. Several controversies remain in terms of incidence, classification, screening, heredity, and treatment [1–3]. The absence of the left circumflex (LCX) artery with a superdominant right coronary artery (RCA) is very rare, and the concomitant anomalous origin of the left anterior descending (LAD) artery turns this association into an unheard, up to now, CAA presentation.

## 2. Case Report

**Figure 1:**
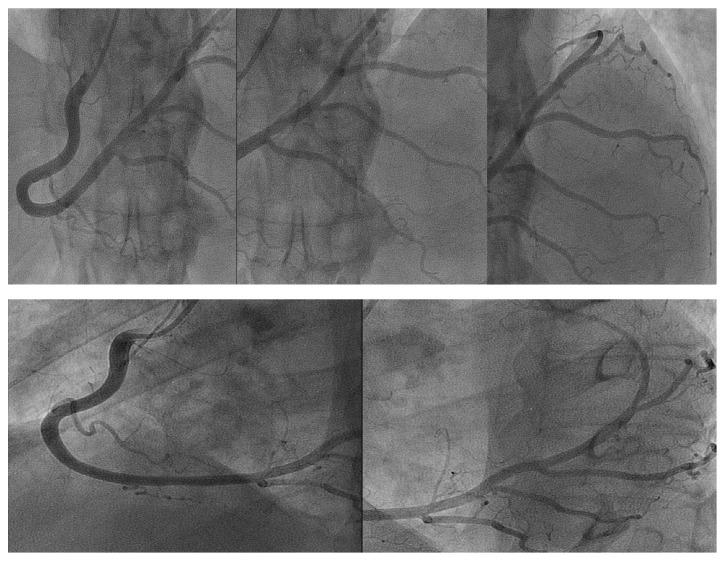
Reconstruction, by subsequent images, of the superdominant RCA, with various posterior descending branches, extending beyond the* crux cordis* and circling the atrioventricular groove, following the expected path of the absent circumflex artery. Superior panel: cranial left anterior oblique view. Inferior panel: left anterior oblique view. RCA: right coronary artery.

**Figure 2:**
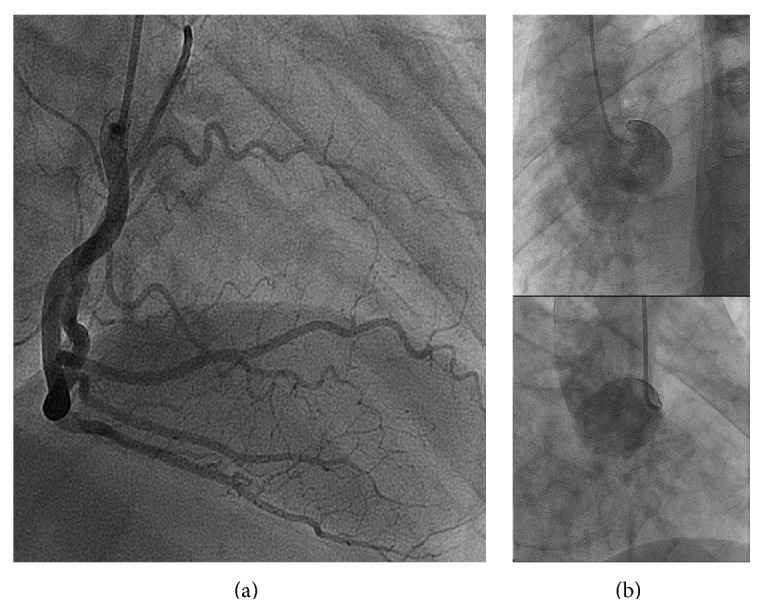
(a) The superdominant RCA in right anterior oblique view. (b) Nonselective injections of contrast media into the left coronary sinus showing no emergent arteries.

**Figure 3:**
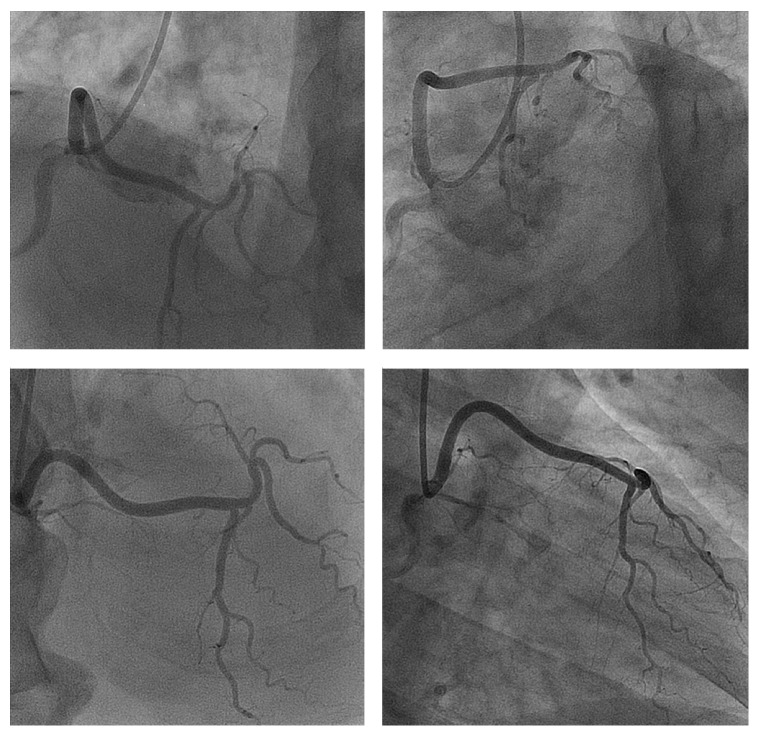
The LCA arising from the right coronary sinus, near to the RCA ostium, and reaching the anterior intraventricular course of the LAD. LCA: left coronary artery; RCA: right coronary artery; LAD: left anterior descending.

A 70-year-old man, active and Caucasian, presented with a three-month history of exertional angina, one episode of syncope after a normal effort, and dyspnea in usual daily activities. There were no previous episodes of myocardial infarction, stroke, coronary artery disease, or personal or familiar histories of sudden cardiac death. The resting electrocardiogram (ECG) showed sinus rhythm and left atrial and ventricular overload. A rest transthoracic echocardiogram revealed preserved left ventricular ejection fraction (0.58, Simpson) and severe aortic valvar stenosis, defined by an orifice area of 0.7 cm^2^ and a transvalvular medium gradient of 50 mmHg. The surgical aortic valve replacement (SAVR) was proposed to him and a preoperative coronary angiogram was, then, requested. The RCA showed a superdominant pattern, with various posterior descending branches, extending beyond the* crux cordis* and circling the atrioventricular groove almost completely, following the expected path of the absent circumflex artery (Figures 1 and 2). A nonselective injection of contrast media into the left coronary sinus revealed no emergent arteries (Figure 2). The left coronary artery, instead, arose from the right coronary sinus, near to the RCA ostium, and reached the anterior intraventricular course of the left anterior descending artery, after passing in front of the pulmonary trunk (Figure 3). There was massive calcification into the aortic valve topography. The systolic transvalvular gradient was 100 mmHg, without intraventricular gradient. The patient is, at the time of this report, waiting the call for the proposed SAVR, in the same functional status, without readmissions or major adverse cardiac or cerebrovascular events.

## 3. Discussion

CAA are congenital changes in their origin, course, and/or structure. Several controversies remain in terms of incidence, classification, screening, heredity, and treatment. Despite being mostly asymptomatic, clinical presentation in adults may result from myocardial ischemia, manifesting as angina, syncope, arrhythmias, and even sudden death. In young athletes, apparently healthy, they are the second most frequent cause of sudden death [3].

Most CAA are discovered as incidental findings during coronary angiographic study or at autopsy with incidence rate of 0.64% to 1.3% reported in the literature [4].

Yamanaka and Hobbs described 126.595 patients undergoing cardiac catheterization from 1960 to 1988 [5]. Separate origins of the LAD and LCX arteries from the left sinus of Valsalva were the most common anomaly, occurring in about 0.41% of the patients studied [4]. Absence of the LCX artery is a very rare congenital anomaly of the coronary circulation in which the artery fails to develop in the left atrioventricular groove, with a few cases reported in the literature [4, 6], and a frequency of only 0.003% according to those authors [5]. In this condition, the inferior, lateral, and posterior walls of the left ventricle are supplied by a superdominant RCA and, sometimes, by a large diagonal branch [4, 5].

If the LCX artery cannot be visualized during angiography, either an ostial total occlusion or congenital agenesis should be suspected. Anomalous origin of the LCX is diagnosed when it is not visualized during left coronary injection in the absence of proximal occlusion, but, instead, it arises separately from the right sinus of Valsalva or as an extension of the RCA [4].

After an extensive review in the pertinent literature, we did not find any report of the herein described association of absence of LCX with superdominant RCA and anomalous origin of the LAD from the right coronary sinus.

In our case, despite the presence of degenerative severe aortic valvar stenosis, no significant lesions were noted in any of the coronary arteries, so the symptoms recently reported by the patient were really due to the progression of the valvar dysfunction.

All interventional cardiologists and cardiac surgeons should be familiar with these anatomic variants since accurate recognition of the course and distribution of the coronary vessels is crucial for proper revascularization strategies in the presence of coronary artery disease [7].

## 4. Conclusion

To the best of our knowledge, this is the first report of this so far unheard coronary anomaly circulation, characterized by the absence of LCX with superdominant RCA and anomalous origin of the LAD from the right sinus of Valsalva.
